# Using mid infrared technology as new method for the determination of the dwell time of salivary substitutes on three dimensional gingiva models

**DOI:** 10.1186/s12901-016-0025-5

**Published:** 2016-03-15

**Authors:** Karin Engelhart, Alice Popescu, Jürgen Bernhardt

**Affiliations:** BioTeSys GmbH, Schelztorstrasse 54-56, D-73728 Esslingen, Germany

**Keywords:** Dry mouth, Dwell time, Gingiva, Mid infrared spectroscopy, New method, Oral care, Salivary substitutes, Sjorgren’s syndrome, Xerostomia

## Abstract

**Background:**

Many people suffer from dry mouth (xerostomia) due to radiotherapy treatment of head and neck cancer, diseases like Sjogren’s syndrome or as adverse effects to prescribed medications. Salivary substitute products like gels or sprays are often used for treatment. Efficacy of those oral care products are regularly assessed by validated or even not validated questionnaires. To determine the adhesion effect over time more objectively a new and sensitive method was established. The following study was designed to assess the dwell time of different oral care products in vitro.

**Method:**

Two different types of surfaces were covered with oral care products and washed using a definite protocol with artificial saliva salt solution. First, oral care gels or oral care sprays were spread to a polystyrene surface of 2.25 cm^2^, then onto cell based three-dimensional gingiva models. The surfaces were washed ten times with artificial saliva salt solution. The resulting washing solutions were examined using mid infrared spectroscopy in order to detect ingredients of the oral care products.

**Results:**

All assessed oral care gels or oral care sprays and their components were detected very sensitive. Even traces of the products were detected in the eluent and thus enabled to differentiate the dwell times of the different products. In general, the dwell time of oral care gels on polystyrene or gingiva models was longer than that of oral care sprays. The use of gingiva models improved the differentiation between different products.

**Conclusions:**

MIR spectroscopy turned out to be a sensitive method to detect salivary substitutes. Differences between single components and different products can be detected. The described method is a simple, reliable and easy process to evaluate the dwell time of oral care products in vitro and thus a useful tool to design optimised salivary substitute products.

**Ethics:**

This is an in vitro study. No ethics or consent was required for this study.

## Background

The subjective sensation of dry mouth, xerostomia, is a symptom that affects many people. Xerostomia can occur due to radiotherapy treatment of head and neck cancer, diseases like Sjögren’s syndrome or as adverse effects to prescribed medications. Sjögren’s syndrome is one of the main cause for xerostomia. Round about 3 % of Americans suffer from Sjögren’s syndrome with 90 % of them being women [1]. Although occurrence of oral cancer is higher in men than in women [2, 3] in total women suffer more often from dry mouth than men. Recent studies of the WHO report that especially for elder people general and associated oral health conditions have a direct influence on quality of life and lifestyle [4] because dry mouth affects eating, talking and increases risk for local infections.

Treatment of xerostomia remains difficult. Salivary production is triggered by parasympathic receptor stimulation for high electrolyte containing salivary as well as sympathic receptor stimulation for salivary proteins [5]. Parasympathomimetic drugs, like pilocarpine hydrochloride, are therefore used for treatment of salivary gland dysfunction but the evidence for successful treatment is limited [6]. Besides non-pharmacological methods like electrostimulation or acupuncture, whose effectiveness remains controversial [7], the most common treatment of dry mouth is the use of salivary substitute products like oral care gels or salivary substitute sprays. But xerostomia recurs as soon as the treatment is interrupted [8]. A long dwell time of oral care products within the oral cavity is therefore one aim in oral care product development. The effectiveness of such products is usually evaluated through semi-quantitative user questionnaires [9, 10]. An objective method is still missing and further in vitro and in vivo studies on properties of saliva substitutes are required [11].

In the present study a new method is presented to evaluate the dwell time of salivary substitute products using mid infrared spectroscopy. Infrared spectroscopy (IR) is a well-established analytical tool in biomedical research [12–14]. An infrared absorption spectrum of a sample records the frequencies of all containing molecules. Each chemical bond in a molecule vibrates at a characteristic frequency depending on the adjacent atoms. Mid infrared (MIR) spectroscopy is mainly used for the analysis of proteins and lipids [15–17]. However, the development of new flow-through cells with pathlength of <10 μm and small sample volumes optimized for the measurement of aqueous solutions [18] enables new applications as it is a fast method for the investigation of molecules in their native state in aqueous solutions very sensitively. With this method even traces of molecules can be detected.

Artificial saliva salt solution shows a characteristic absorption spectrum. Since saliva substitute products mimic the saliva, the absorption spectrum resembles that of the salt solution. As the applied saliva substitute products contain additional care ingredients their spectra should be clearly distinguishable from the saliva salt solution through specific additional peaks.

Our in vitro study aimed to develop an assay for the objective measurement of the dwell time of oral care products. The tested products were applied onto both synthetic surface and cellular gingiva models and removed by a defined washing procedure. The washing solutions were analysed for traces of the products using MIR technology. This procedure simulates the wash-out of the care products in the mouth by natural saliva flow. Differences in the dwell time of different saliva substitute product types (gels and sprays) were examined on artificial and cellular surfaces.

## Methods

### Material

#### Chemicals and cell lines

Sodium bicarbonat, sodium chloride and potassium chloride were purchased from Merck (Darmstadt, Germany). All oral care products (Aldiamed Gel, Aldimed Spray (both Certmedica), biotène Oral Balance (GSK), BioXtra Gel (GUM), Glandosan (CellPharma), Saliva Natura (Parnell Pharmaceuticals)) were purchased in a local pharmacy. Polystyrene multi-well plates were from Greiner BioOne (Frickenhausen, Germany).

Artificial saliva salt solution was composed of 4,2 g/L sodium bicarbonate, 0,5 g/L sodium chloride, and 0,2 g/L potassium chloride dissolved in aqua bidest. The pH was adjusted to 7,3.

Three-dimensional human gingiva model (Epi-Gin) and maintenance medium were from MatTek (Ashland MA, USA).

### Method

#### Dwell time assay on polystyrene surface

In 6-well multi well plates 100 μL of each oral care product was applied onto a marked area of 1,5 cm × 1,5 cm and allowed to air dry for 10 min. The product was rinsed off with 1500 μL of artificial saliva salt solution by slightly shaking on an orbital shaker. The washing solution was withdrawn for analytics. This washing step was repeated ten times, each washing solution was collected separately. All washing solutions were directly analysed using mid infrared spectroscopy. The experiments were performed in triplicates.

#### Dwell time assay on organotypic model

On top of three-dimensional human gingiva models (Epi-Gin; MatTek, Ashland MA, USA;) 25 μL of each oral care product was applied and allowed to air dry for 10 min. The product was rinsed off with 400 μL of artificial saliva salt solution. The washing solution was withdrawn for analysis. This washing step was repeated ten times, each washing solution was collected separately. All washing solutions were directly analysed using mid infrared spectroscopy. The experiments were performed in triplicates.

#### Analytical method

The washing solutions were directly analysed using an AquaSpec™ MIRA-LAB LHS-500analyser. AquaSpec™ spectroscopy is based on mid infrared spectroscopy in a spectral range of 3001–951 cm^−1^ [18]. Absorption spectra and PCA analysis were used for evaluation.

## Results and discussion

The aim of this project was to evaluate the maximal adhesion, mean dwell time, of the respective oral care product to the surface. A polystyrene surface was used as first proof of principle for the analytical method. Subsequently, in order to be as close as possible to in vivo situation the oral care products were applied onto the cellular surface of three-dimensional gingival models.

**Fig. 1:**
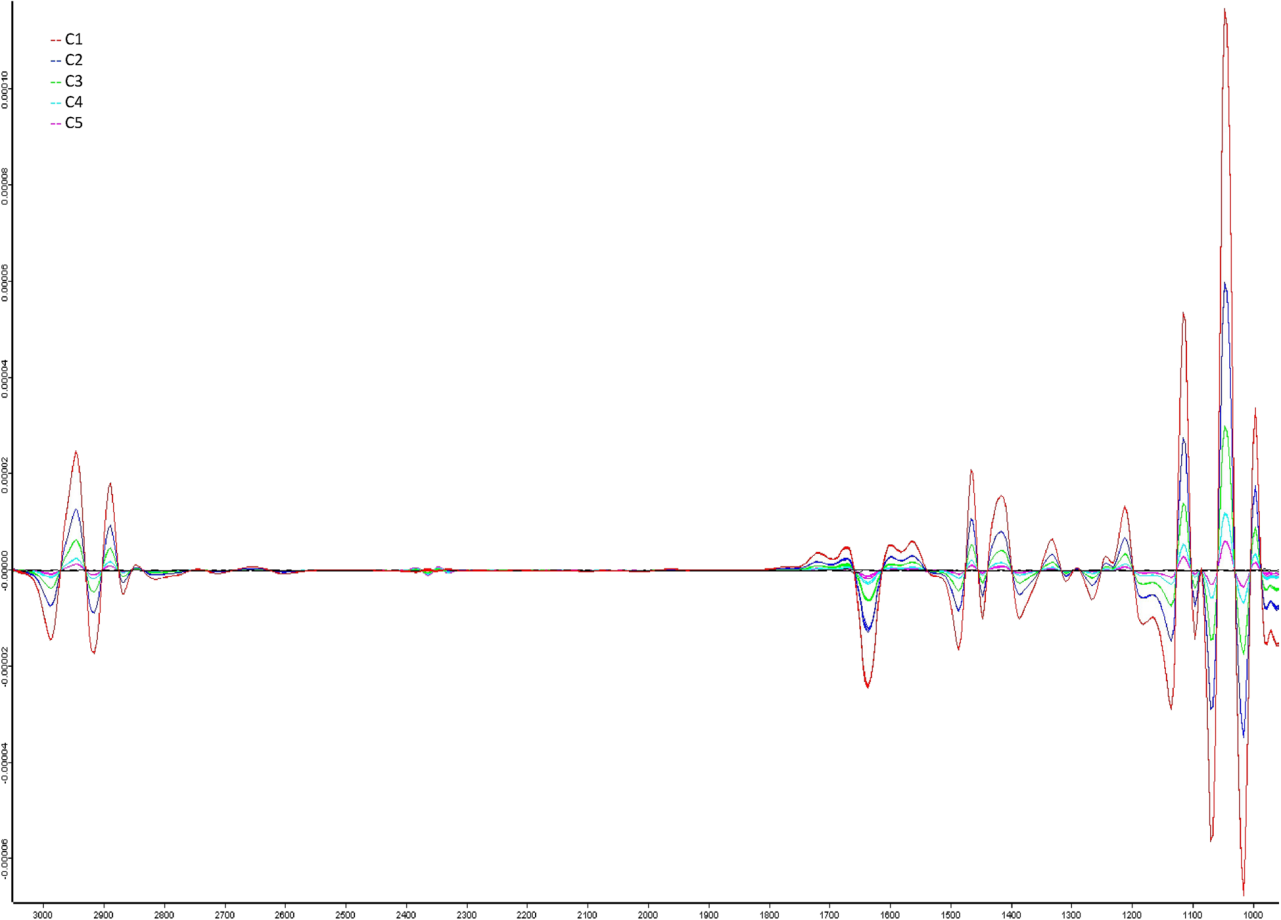
Sensitivity of the analysis method. Absorption spectrum of aldiamed gel in five dilutions (C1 = 1:25; C2 = 1:50; C3 = 1:100; C4 = 1:250; C5 = 1:500)

The MIR technology is a very sensitive analytical method for the detection of the components in aqueous solutions [18–21]. Each of the tested oral care products showed a characteristic absorption spectrum depending on its own ingredients and it was able to differentiate the tested products from the artificial salivary salt solution used as washing solution. A dilution series showed that the test product can be distinguished from the salivary salt solution used as eluent even in very low concentrations, for the oral care gel aldiamed for example a dilution of 1:500 (see Fig. 1) can be determinated.

**Fig. 2:**
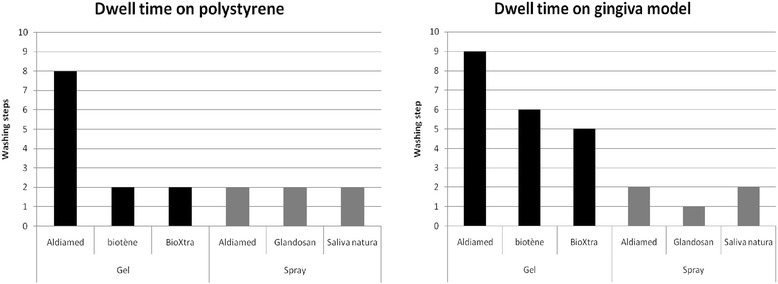
Dwell time of the different salivary substitute products on polystyrene and cellular gingival model (*n* = 3, mean ± SD)

Dwell time experiments showed that the method is capable of differentiating adherence between different product types. The oral care gels adhere longer to the surfaces than oral care sprays. The experiments showed also that the gingival models allow for a better differentiation between the single products than the polystyrene. On the polystyrene surface, differentiation was only possible for the oral care gels, yet no differentiation between the spray products was seen (see Fig. 2). Components of all oral care sprays were only detectable in the first two washing solutions. Components of biotène gel and bioXtra gel were found in the first two washing solutions where as components of the aldiamed gel were detectable up to the eighth washing solution.

**Fig. 3:**
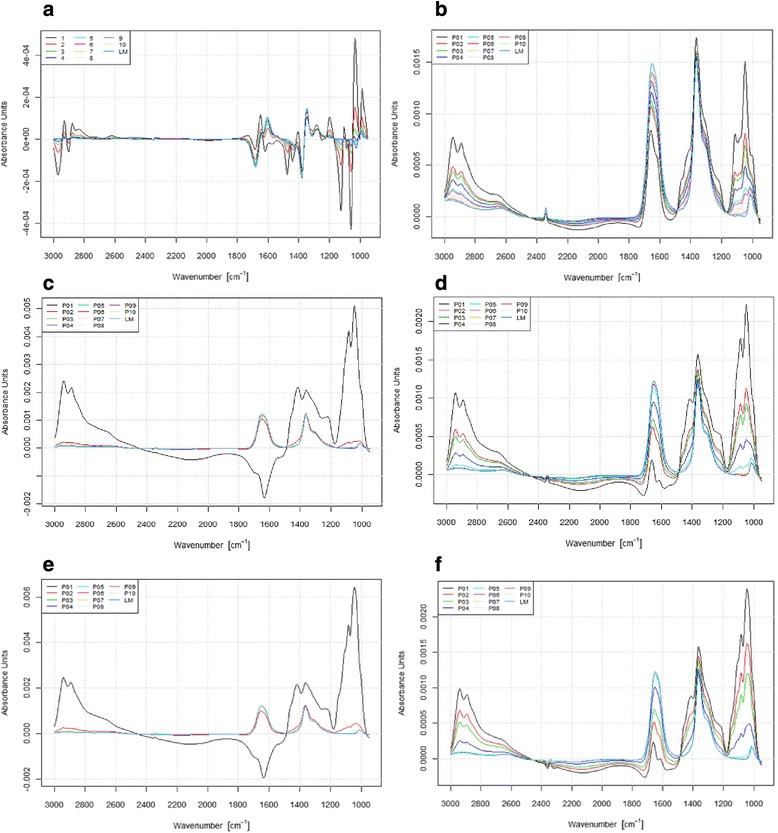
Absorption spectra of the solutions after the single rinsing steps (P01-P10) after application of aldiamed gel on polystyrene (**a**, **c**, **e**) and cellular gingival model (**b**, **d**, **f**)

In contrast, when using the cellular gingival models it was possible to differentiate between all products tested. Especially for the oral care gels a clear difference in the dwell time of the products was seen. The oral care gels biotène gel and bioXtra gel disappeared after the sixth and fifth washing step, respectively, and thus remained longer on the cellular surface than on the polystyrene surface. The aldiamed gel could not be longer detected after the eighth washing step which did not differ from that found for the polystyrene surface (see Fig. 3). Even between the oral care sprays a difference was detected when using gingival model as surface. The small difference in the dwell time between the products was similar in all repeated measurements and thus highly reproductive.

**Fig. 4:**
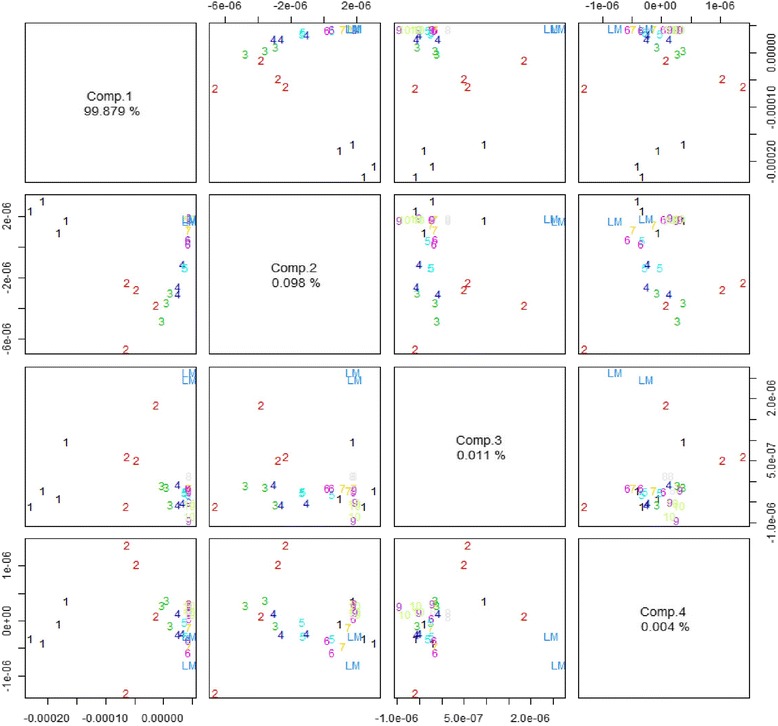
PCA analysis of the four main components of aldiamed gel in the washing steps 1–10 and pure washing solution (LM)

In the present study it was not the aim to identify single components of the oral care products but to detect traces of the product in the washing solution. However, using PCA analysis together with AquaSpec™ technology it was possible to distinguish between different fractions (see Fig. 4). One major component was attributed to ingredients with high OH-group content and a second fraction to such rich in COOH-groups. It was shown that these two main components were rinsed off differently. The first fraction, rich in OH-groups, was washed off continuously over the first five washing steps. The second fraction, rich in COOH-groups, was more or less stable in the first four washing steps and disappeared in washing step seven.

Using mid infrared spectroscopy seems to be a very sensitive method to detect the adhesion properties of the salivary substitutes. It is not necessary to use a specific analytical method, e.g. a HPLC method, for one or several components of the product. MIR gives an overall spectrum of the whole product and thus its successive disappearance can be monitored very well. This advantage of the mid infrared spectroscopy over often time-consuming and expensive current methods was also described for the detection and identification of household fungi [22], characterization of wine [23], and in clinical analysis [24].

## Conclusions

In conclusion, mid infrared spectroscopy based AquaSpec™ technology proved to be a valuable tool for the analysis of aqueous solutions. With this special technique limitations of IR spectroscopy especially in aqueous systems have been overcome and it is a very rapid and sensitive method for the detection of complex matrices.

We have been able to detect the tested products even in dilutions up to 1:500. The reproducibility of the analysis was very good. Using the absorption spectra we were able to differentiate the dwell time of the different products even within a product type. As supposed from viscosity of the products the dwell time of the gels was longer whereas the sprays were washed off immediately. The use of three-dimensional gingival models, a cellular surface, provided a surface similar to the in vivo situation and improved adhesion properties and differentiation between the products. Surprisingly, the dwell time for the sprays did not prolong on the gingival model compared that on the polystyrene surface - this might be due to the very low viscosity of these products - but the differentiation between different products was refined. In this study the dwell time of some oral care products was assessed in vitro. It was not the aim to trace single components and their adhesion properties. But as seen for COOH- rich and OH-rich components this could be also possible and adhesion properties might be different.

In our study MIR spectroscopy in combination with three-dimensional gingival models has been shown to be a very sensitive method to detect the adhesion properties of the salivary substitutes in vitro. It is not necessary to use a specific analytical method, e.g. a HPLC method, for one or several components of the product. IR spectroscopy gives an overall spectrum of the whole product and thus its successive disappearance can be monitored very well. Furthermore this method could be a first step in investigations to uncover oral care products/saliva substitutes with short dwell times resulting in no relevant improvement in quality of life.

## Abbreviations

IR: infrared
MIR: mid infrared
PCA: principal component analysis
